# Synthesis, Characterization and *in vitro* Studies of a Cathepsin B‐Cleavable Prodrug of the VEGFR Inhibitor Sunitinib

**DOI:** 10.1002/cbdv.201800520

**Published:** 2018-12-19

**Authors:** Claudia Karnthaler‐Benbakka, Bettina Koblmüller, Marlene Mathuber, Katharina Holste, Walter Berger, Petra Heffeter, Christian R. Kowol, Bernhard K. Keppler

**Affiliations:** ^1^ Institute of Inorganic Chemistry, Faculty of Chemistry University of Vienna Waehringer Straße 42 AT-1090 Wien; ^2^ Institute of Cancer Research and Comprehensive Cancer Center Medical University of Vienna Borschkegasse 8 A AT-1090 Wien; ^3^ Research Cluster ‘Translational Cancer Therapy Research' University of Vienna and Medical University of Vienna AT-1090 Wien

**Keywords:** tyrosine kinase inhibitor, prodrugs, inhibitors, cathepsin B, sunitinib, vascular endothelial growth factor receptor (VEGFR)

## Abstract

Since several decades, the prodrug concept has raised considerable interest in cancer research due to its potential to overcome common problems associated with chemotherapy. However, for small‐molecule tyrosine kinase inhibitors, which also cause severe side effects, hardly any strategies to generate prodrugs for therapeutic improvement have been reported so far. Here, we present the synthesis and biological investigation of a cathepsin B‐cleavable prodrug of the VEGFR inhibitor sunitinib. Cell viability assays and Western blot analyses revealed, that, in contrast to the non‐cathepsin B‐cleavable reference compound, the prodrug shows activity comparable to the original drug sunitinib in the highly cathepsin B‐expressing cell lines Caki‐1 and RU‐MH. Moreover, a cathepsin B cleavage assay confirmed the desired enzymatic activation of the prodrug. Together, the obtained data show that the concept of cathepsin B‐cleavable prodrugs can be transferred to the class of targeted therapeutics, allowing the development of optimized tyrosine kinase inhibitors for the treatment of cancer.

## Introduction

With the aim of overcoming common drawbacks of chemotherapeutic agents, such as a low therapeutic index due to high toxicities and side effects and a lack of tumor selectivity, the prodrug concept has attracted considerable attention in the field of cancer research since the 1950s.^1^ In general, this concept describes a pharmacologically inert derivative of a drug that can be converted to its active analogue *in vivo* by enzymatic or non‐enzymatic mechanisms,^2^ which, in the case of anticancer drugs, should preferably be tumor‐specific. A broad spectrum of different targeting strategies has been developed in the last decades.^3^, ^4^, ^5^ One of them is the activation *via* cleavage by cathepsin B, a (usually lysosomal) cysteine protease, which is overexpressed and secreted to the cell surface and pericellular space by a wide variety of cancer types.^6^ High levels of cathepsin B are known to strongly contribute to the invasiveness of malignant cells, and expression as well as activity of cathepsin B have been correlated with worse prognosis in the clinic.^7^,^8^ This tumor‐specific overexpression of cathepsin B can be exploited for the design of prodrugs carrying a cathepsin B‐cleavable entity, resulting in the release of the active species only in the surrounding of tumor cells that express cathepsin B on their cell surface. One example for the successful use of this enzymatic activation is brentuximab vedotin (*Adcetris*®), an antibody‐drug conjugate that has already been approved for the treatment of relapsed or refractory *Hodgkin*’s lymphoma and relapsed or refractory systemic anaplastic large cell lymphoma.^9^ In addition, a range of other antibody conjugates as well as doxorubicin prodrugs using cathepsin B cleavage as activation mechanism are under (pre)clinical evaluation.^10^, ^11^, ^12^, ^13^, ^14^ Interestingly, most of the so far used drugs for the design of cathepsin B‐cleavable therapeutics are either conventional small‐molecule chemotherapeutics or natural bacterial toxins.^5^ Thus, to the best of our knowledge, for the large class of small‐molecule tyrosine kinase inhibitors (TKIs), no such approach has been reported so far.

Within this study, we synthesized and investigated cathepsin B‐cleavable prodrugs of the vascular endothelial growth factor receptor (VEGFR) inhibitor sunitinib (Sutent®), which is approved for the treatment of gastrointestinal stromal tumors (GIST) after failure of imatinib treatment, advanced renal cell carcinoma (RCC), and unresectable or metastatic pancreatic neuroendocrine tumors (pNET).^15^ Despite the belief that targeted therapeutics (including sunitinib and other representatives of the class of TKIs) should possess a much higher tolerability than conventional chemotherapeutics, their clinical use is limited due to severe side effects, such as hypertension, hepatotoxicity, hypothyroidism, hand‐foot syndrome, papulopustular rash, gastrointestinal perforation, *etc*.^16^,^17^ Thus, also for TKIs, a strategy to circumvent these problems would be a major clinical advantage. In this work, we designed cathepsin B‐activatable prodrugs of the VEGFR inhibitor sunitinib, since studies suggested a role of cathepsin B in inducing angiogenesis.^6^,^18^, ^19^, ^20^ This would offer the possibility of targeting tumor sites which comprise high levels of both: cathepsin B and VEGFR.

**Figure 1:**
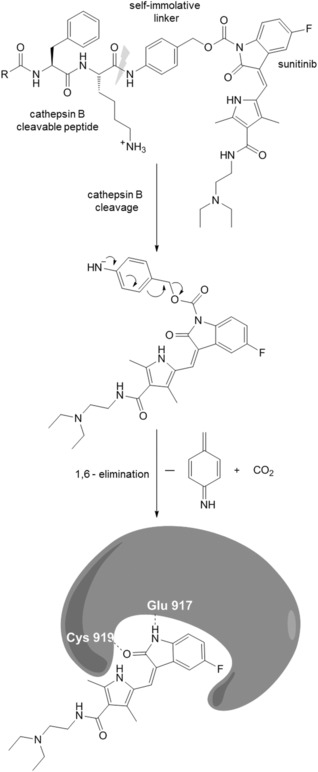
Schematic overview of the cathepsin B‐cleavable prodrug concept for sunitinib. Hydrogen bonds are indicated by dashed lines.

In order to generate a prodrug of a TKI, the compound has to be inactivated by derivatizing a position essential for its tyrosine kinase inhibiting potential. Only after activation through cathepsin B cleavage, the unmodified drug should be released. In the case of sunitinib, the oxindole nitrogen is a suitable site for derivatization, as co‐crystal structures of VEGFR2 and sunitinib revealed a crucial hydrogen bond between this nitrogen and Glu917 in the adenine pocket.^21^ The cathepsin B‐cleavable unit was adopted from *Dubowchik et al*.^22^,^23^ comprising the Z‐protected dipeptide Phe‐Lys, followed by a self‐immolative *p*‐aminobenzylcarbonyl (PABC) linker. Upon cathepsin B cleavage of the dipeptide, the PABC‐linker is supposed to undergo 1,6‐elimination with subsequent release of the active drug (the schematic overview of this concept is outlined in *Figure* 1). The Phe‐Lys dipeptide has demonstrated its efficacy with superior properties compared to other peptides (often tetrapeptides) commonly used as drug carrier linkers.^22^ In addition, reduced toxicity and promising activity against different cancer types was recently reported for Ac‐Phe‐Lys‐PABC‐doxorubicin *in vitro* and *in vivo*.^24^, ^25^, ^26^

## Results and Discussion

### *Synthesis*

**Scheme 1:**
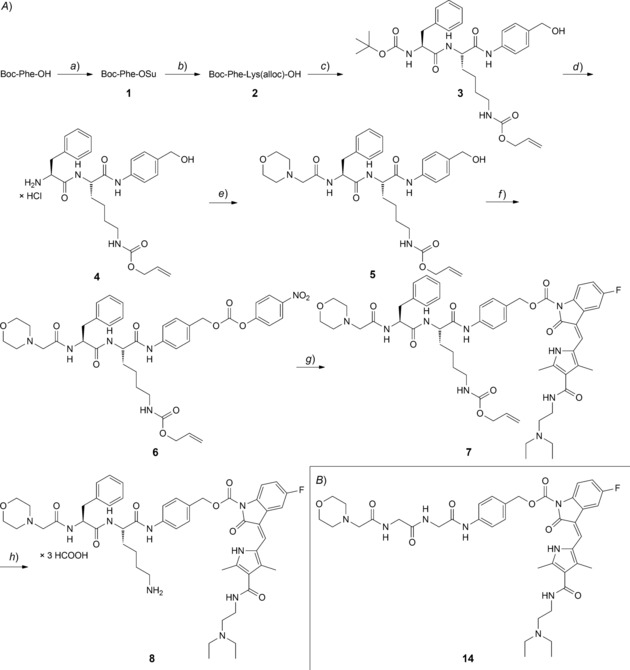
*A*) Synthetic route of prodrug **8**. Reagents and conditions: *a*) NHS, DCC, THF abs., 0 °C to room temperature; *b*) H‐Lys(alloc)‐OH, NaHCO_3_, DME, H_2_O; *c*) PABOH, EEDQ, THF; *d*) HCl conc., EtOH; *e*) morpholin‐4‐yl‐acetic acid, EEDQ, Et_3_N, THF/EtOH; *f*) bis(4‐nitrophenyl) carbonate, DIPEA, DMF abs.; *g*) sunitinib, 4‐DMAP, DMF abs.; *h*) Pd(PPh_3_)_4_, NDMBA, DMF abs. *B*) Chemical structure of the Gly‐Gly reference compound **14**.

The synthesis of Z‐Phe‐Lys(alloc)‐PABC‐PNP was performed by a straightforward four‐step procedure from *Dubowchik et al*.^23^ Sunitinib was then introduced using 4‐DMAP in dry DMF with reaction times of up to seven days followed by TLC. Alloc deprotection was effected by treatment with Pd(PPh_3_)_4_ and, as a less toxic hydride source than Bu_3_SnH, Me_2_NH⋅BH_3_ (40 equiv.). Unfortunately, the final compound Z‐Phe‐Lys‐PABC‐Sun was not soluble at all in aqueous solution at acceptable concentrations, not even by the addition of 10 % DMSO. Thus, we decided to introduce morpholine as water‐solubilizing group at the peptide *N*‐terminus. To this aim, Boc‐Phe‐OH was used as starting material, which was converted to Boc‐Phe‐Lys(alloc)‐PABOH (**3**) similarly to *Dubowchik*’s method.^23^ After cleavage of the Boc‐protecting group, morpholin‐4‐yl‐acetic acid was introduced to give Morph‐Phe‐Lys(alloc)‐PABOH (**5**). Attempts to generate the PNP‐active ester using PNP chloroformate as used in the case of the Z‐protected derivative failed, likely due to an attack of the morpholine nitrogen on the acid chloride. Alternatively, **5** was treated with bis(4‐nitrophenyl) carbonate and DIPEA in dry DMF to give the desired activated cathepsin B‐cleavable unit **6**. After introduction of sunitinib, also the deprotection step had to be adjusted, resulting in the use of Pd(PPh_3_)_4_ and 1.1 equivalent of NDMBA (1,3‐dimethylbarbituric acid) as hydride source. Final purification through RP‐HPLC yielded the desired product Morph‐Phe‐Lys‐PABC‐Sun ⋅ 3 HCOOH (**8**) (*Scheme* 1 *,a*). As a reference compound, Morph‐Gly‐Gly‐PABC‐Sun (**14**; *Scheme* 1 *,b*), which contains the dipeptide sequence Gly‐Gly that should not be cleaved by cathepsin B, was prepared in a similar way (*Scheme S1*).

### *Selection of Cell Lines*

To allow the biological testing of our novel drugs, an appropriate cell line panel of sunitinib‐sensitive cell lines with different cathepsin B expression levels needed to be selected. To this end, a collection of 15 cancer cell models originating from different tissues (including diverse renal cell carcinomas) were tested for their sunitinib sensitivity, sunitinib target expression (VEGFR‐1 and PDGFR *β*) as well as cathepsin B protein expression (by Western blotting) and activity (by zymography). A summary of the collected data is shown in *Table* 1 (see also *Figures S11*–*S13*, *Supplementary Information*). Based on this data, the cell lines Caki‐1 (strong cathepsin B expression and activity), RU‐MH (strong cathepsin B expression and activity) and HCT116 (weak cathepsin B expression and activity) were selected for the subsequent biological testing of the drugs.

**Table 1:** Overview of sunitinib sensitivity, sunitinib target receptor tyrosine kinase (RTK) expression, and cathepsin B status of the used cell lines.

Cell line	Sunitinib		RTK Expression	Cathepsin B status	
	*IC*_50_ [μm]	SD		Western blot	Zymo‐ graphy
Calu3	9.6	0.1	VEGFR‐1	+++	–
H1703	0.5	0.0	VEGFR‐1/PDFGR *β*	–	–
H1975	>10	–	n.t.^[a]^	n.t.	+
HCC827	5.2	0.5	VEGFR‐1	+	+
U87MG	>10	–	n.t.	n.t.	n.t.
Caki‐1	6.8	0.2	VEGFR‐1	+++	+++
Caki‐2	>10	–	–	+	–
ORMW/8	7.9	0.7	VEGFR‐1	+++	+++
RU‐MH	6.6	1.3	VEGFR‐1	+++	++
A431	7.3	0.4	VEGFR‐1	++	+++
A375	4.4	1.0	n.t.	–	–
PC‐3	8.7	0.1	n.t.	–	–
HCT116	4.6	0.3	VEGFR‐1	+/–	–
A549	7.2	0.0	n.t.	+/–	++
H520	4.2	0.4	VEGFR‐1	+	n.t.

^[a]^ n.t. – not tested

### *Cytotoxicity Tests of Compounds* **8** *and* **14**

**Figure 2:**
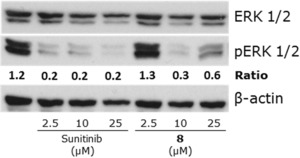
Inhibitory effects of prodrug **8** in comparison to sunitinib on RTK signaling. Caki‐1 cells were grown in medium with FCS and treated with the indicated drug for 4 h. Then, they were harvested and lysed, and the impact on ERK phosphorylation as readout for RTK signaling was analyzed by Western blotting.

After 72 h exposure, viability tests revealed that our new sunitinib prodrug **8** had similar but about 1.1 to 1.5‐fold reduced anticancer activity compared to free sunitinib (*Table* 2, *Figure S14*). In contrast, the reference drug **14**, which should not be cleaved by cathepsin B, revealed distinctly less activity indicating that the attachment of the Morph‐Gly‐Gly‐PABC‐unit indeed reduced sunitinib activity. Moreover, subsequent Western blot analysis (*Figure* 2) indicated that after 4 h of drug treatment **8** was able to inhibit phosphorylation of ERK (a VEGFR‐1‐downstream protein, which is frequently used as read‐out for activity of this signaling pathway^27^,^28^). Comparable to the viability assays, **8** was less active (full inhibition at 10 μm) than free sunitinib (full inhibition at 2.5 μm), which can be explained by the required drug cleavage before the onset of drug activity, while the free drug is already capable of full anticancer activity. However, in contrast to the expectations, HCT116 cells, which were characterized by only weak cathepsin B expression and activity, were sensitive to our new prodrug comparable to the high cathepsin B‐expressing Caki‐1 and RU‐MH cells. Possibly already low levels of cathepsin B are sufficient to cleave the prodrug unit or other enzymes can also activate the compound.

**Table 2:** *IC*_50_ values of the tested cell lines after treatment with the indicated drugs for 72 h.

Cell line	Sunitinib		**8**		**14**	
	*IC*_50_ [μm]	SD	*IC*_50_ [μm]	SD	*IC*_50_ [μm]	SD
HCT116	5.2	1.8	7.8	1.1	14.9	5.4
Caki‐1	7.9	1.8	12.2	2.7	>20	–
RU‐MH	6.9	0.5	7.9	0.3	18.1	7.9

### *Cathepsin B Cleavage Assays and Activation Studies*

**Figure 3:**
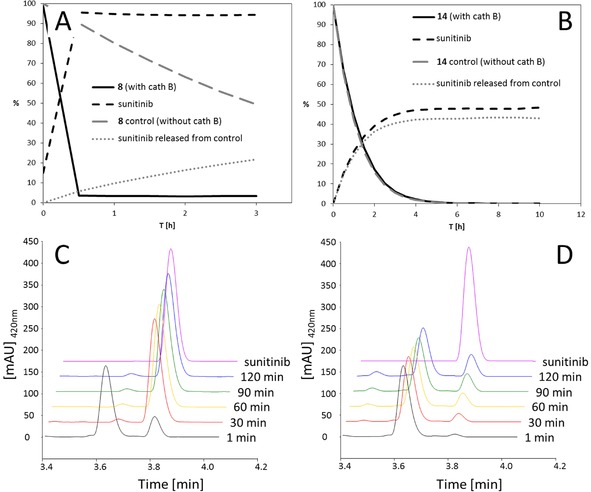
Cathepsin B assay. A), B): Time curves of (A) compound **8** and (B) compound **14** after incubation with and without (‘control’) human liver cathepsin B at pH 5 and 37 °C. C), D): Chromatograms of the incubation study of compound **8** (C) with human liver cathepsin B, and (D) without cathepsin B.

Consequently, to better understand the activation of our new prodrug, cathepsin B cleavage assays were performed with **8** and **14** to demonstrate proof of principle for this concept. To this end, the substrates were incubated with human liver cathepsin B at pH 5.0 and 37 °C, and the reaction was followed by HPLC. In order to distinguish between the effects of enzymatic cleavage and hydrolysis, incubation of the compounds without cathepsin B under the same conditions (denoted as ‘**8** control’ and ‘**14** control’ in *Figure* 3) served as a reference experiment. As *Figure* 3 *,A*, *3,C*, and *3,D* shows, compound **8** was readily activated by cathepsin B with a half‐life of <15 min resulting in release of sunitinib via self‐immolation of the PABC linker. By contrast, as expected, for the reference compound **14** no difference could be observed between the measurements with/without cathepsin B, identifying it as the desired non‐cathepsin B‐cleavable negative control (*Figure* 3 *,B*).

**Scheme 2:**
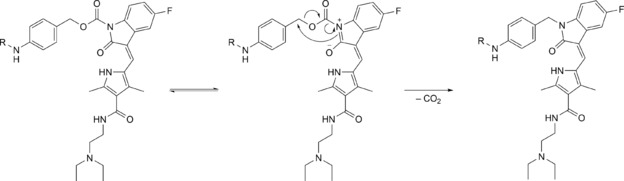
Postulated mechanism of the rearrangement of the enol form of **8** and **14** from carbamate to direct linkage in aqueous solution.

However, the activation studies also revealed an unexpected hydrolytic lability for both compounds. Thus, additional stability measurements in 10 mm sodium phosphate buffer, pH 7.4, were performed with **8** and **14** at 37 °C, monitored by reversed‐phase HPLC chromatography. As observed before, both showed fast hydrolysis with half‐lives of *ca*. 2 h and *ca*. 30 min, respectively, and only few percent of remaining substrate after 24 h (*Figures S5–S6*, *Supplementary Information*). Since *Dubowchik* stated that the corresponding doxorubicin derivative was even stable in human plasma at 37 °C,^23^ the reason for the lability of **8** and **14** has to be the carbamate linkage to the lactam nitrogen. In fact, Morph‐Phe‐Lys‐PABOH was detected by HPLC‐MS measurements as main fragment of **8**, indicating hydrolysis at this position. Notably, in the literature, substances with a similar substitution at the oxindole nitrogen were investigated without mentioning any stability problems.^29^,^30^ Thus, it has to be questioned, whether only our particular design is responsible for the low stability or whether this problem just has not been recognized so far. Moreover, also fragments other than free drug were formed in both cases (*Figures S7–S8*, *Supplementary Information*). Especially for **14**, an intense signal was observed, that could be assigned to the mass of the directly linked *p‐*aminobenzyl unit to the sunitinib oxindole nitrogen. In fact, the presence of such a rearrangement product that was also observed for **8** (however, with a much lower intensity) could be confirmed by high resolution mass spectrometry. The postulated mechanism of this rearrangement that implies a keto‐enol equilibrium is initiated by an attack of the nitrogen double bond on the PABC moiety, followed by the elimination of CO_2_ (*Scheme* 2). The amount of this directly linked derivative, which was stable over a period of 24 h according to HPLC‐MS studies, also explains the rather low percentage of hydrolytic sunitinib release (only 30–40 %, *Figures S5–S6*, *Supplementary Information*). Notably, further stability experiments revealed that the rearrangement product of both **8** and **14** was formed by an even higher extent in medium (*Mc Coy's*) with 10 % fetal calf serum than in phosphate buffer (*Figures S9–S10*, *Supplementary Information*). As we assume that the cytotoxicity of this directly linked derivative is quite low (no release of sunitinib), this might be the reason for the reduced cytotoxic activity of the non‐cathepsin B‐cleavable compound **14** in viability assays, despite its partial hydrolysis. For compound **8** (with much lower tendency to form the rearrangement product) the activity in Caki‐1 and RU‐MH cells could be explained by cathepsin B cleavage and subsequent self‐immolation with release of free sunitinib. However, in case of HCT116 cells with only weak cathepsin B expression, it is still uncertain whether the activity of **8** is caused by prodrug hydrolysis, if low levels of cathepsin B are sufficient to cleave the prodrug unit, or whether another enzyme activates the compound.

## Conclusions

In this study, a cathepsin B‐cleavable prodrug of the VEGFR inhibitor sunitinib together with a non‐cleavable control, both containing a water‐soluble unit, have been successfully synthesized through a multi‐step procedure. Their antiproliferative potency was tested against suitable sunitinib‐sensitive cancer cell lines which were previously evaluated for their VEGFR‐1, PDGFR *β*, as well as cathepsin B expression and cathepsin B activity by Western blot analysis and zymography, respectively, showing similar activity of **8** compared to the original drug sunitinib, while the non‐cleavable reference drug **14** was distinctly less active. Moreover, a cathepsin B cleavage assay confirmed the desired enzymatic activation of **8** and inactivity of **14**, showing proof of principle for the first time for this prodrug concept for the class of targeted therapeutics. However, also hydrolytic lability of the compounds with the formation of stable rearrangement products was observed, complicating the interpretation of the obtained biological data, especially regarding the response of the only weak cathepsin B‐expressing HTC116 cells. Thus, future investigations with altered linker moieties will be performed to optimize the applicability of cathepsin B‐based activation approaches for targeted therapeutics. Moreover, the *in vivo* evaluation of this strategy for tumor‐specific drug activation is necessary.

## **Experimental Section**

### *General*

Mass spectrometry was performed on a *Bruker HCT plus ESI‐QIT* spectrometer, high resolution spectra were obtained from a *Bruker maXis ESI‐Qq‐oaRTOF* spectrometer. Expected and experimental isotope distributions were compared. ^1^H‐ and ^13^C‐NMR spectra were recorded in (D_6_)DMSO, CDCl_3_, and/or MeOD, referring to the respective solubility of the synthesized compounds, with a *Bruker Avance III* 500 MHz spectrometer at 500.32 (^1^H) and 125.81 (^13^C) MHz at 298 K, with chemical shifts referenced to the solvent residual peak as an internal standard. NMR Data are reported indicating the chemical shift (*δ*, [ppm]), the multiplicity (*s*, *singlet*; *d*, *doublet*; *t*, *triplet*; *q*, *quartet*; *m*, *multiplet*, *etc*.), the coupling constant (*J*, [Hz]), and the integration. All final compounds displayed ≥95 % purity as determined by elemental analysis performed by the Microanalytical Laboratory of the University of Vienna. Stability and activation upon cathepsin B cleavage were determined by RP‐HPLC on a *Dionex UltiMate 3000 UHPLC* system controlled by Chromeleon 6.8 chromatography software. The experimental conditions were as follows: stationary phase: ethylene bridged hybrid *C18*; column: *Acquity UPLC BEH C18*, 130 Å, 1.7 μm, 3.0 mm×50 mm (*Waters Corp*., Massachusetts, USA); column temperature: 25 °C; mobile phase: H_2_O/MeCN (0.1 % HCOOH), HPLC grade; flow rate: 0.6 ml/min, injection volume: 10 μL; gradient: 5–95 % MeCN in 6 min. Decomposition products were identified through HPLC‐MS on a 1*260 Infinity Bio‐Inert LC* System from *Agilent Technologies*, controlled by an *Agilent* OpenLAB CDS ChemStation software (Edition Rev. C.01.06), coupled to an *amaZon SL* Ion Trap mass spectrometer with HyStar 3.2 and Data Analysis 4.0 software package (*Bruker Daltonics*).

### *Chemicals and Materials*

All solvents and reagents were obtained from commercial suppliers and used without further purification. Anhydrous solvents were bought from *Fisher Scientific GmbH* (Austria) over molecular sieves. Sunitinib was purchased from *LC Laboratories*®, *N* ^*ϵ*^‐allyloxycarbonyl‐l‐lysine from *Chem‐Impex International, Inc*., and morpholin‐4‐yl‐acetic acid from *Activate Scientific GmbH*.

### *Preparation of Compounds*

**Boc‐Phe‐OSu** (**1**).^31^ A solution of Boc‐Phe‐OH (2.00 g, 7.54 mmol) and *N*‐hydroxysuccinimide (954 mg, 1.1 equiv.) in dry THF (15 mL) at 0 °C was treated with DCC (1.63 g, 1.05 equiv.). The resulting mixture was allowed to warm to room temperature. After 23 h the solid byproduct was filtered off, washed with THF, and the filtrate was evaporated. The residue was dissolved in CH_2_Cl_2_ (20 mL), allowed to stand for 1 h, and filtered off again. Evaporation of the filtrate yielded the product as a white powder (2.58 g, 94 %). ^1^H‐NMR (MeOD): 1.39 (*s*, 9 H); 2.87 (*s*, 4 H); 3.05 (*dd*, *J*=14, 10, 1 H); 3.32–3.36 (*m*, 1 H); 4.74–4.79 (*m*, 1 H); 7.24–7.35 (*m*, 5 H).

**Boc‐Phe‐Lys(alloc)‐OH** (**2**).^32^ A mixture of H‐Lys(alloc)‐OH (1.70 g, 7.48 mmol) and NaHCO_3_ (628 mg, 1 equiv.) in H_2_O (25 ml) was added to a stirred solution of **1** (2.58 g, 0.95 equiv.) in DME (25 ml) which was cooled in a cold water bath. The resulting mixture was allowed to warm to room temperature. After 23 h a small amount of solid was filtered off, and the filtrate was diluted with H_2_O (42 mL). The solution was acidified to pH 3 with 15 % aqueous citric acid and extracted with AcOEt (3×75 mL). The combined organic layers were washed with H_2_O and brine (100 mL each), dried over MgSO_4_, filtered, and concentrated *in vacuo* to give a white solid (2.95 g, 87 %). ^1^H‐NMR (MeOD): 1.39 (*s*, 9 H); 1.39–1.46 (*m*, 2 H); 1.51–1.58 (*m*, 2 H); 1.71–1.79 (*m*, 1 H); 1.87–1.94 (*m*, 1 H); 2.84 (*dd*, *J*=14, 9, 1 H); 3.12–3.18 (*m*, 3 H); 4.36 (*dd*, *J*=9, 5, 1 H); 4.42 (*dd*, *J*=9, 5, 1 H); 4.53 (*d*, *J*=5, 2 H); 5.19 (*d*, *J*=11, 1 H); 5.30 (*d*, *J*=17, 2 H); 5.90–5.98 (*m*, 1 H); 7.22–7.31 (*m*, 5 H).

**Boc‐Phe‐Lys(alloc)‐PABOH** (**3**).^32^ To a solution of **2** (2.95 g, 6.18 mmol) and PABOH (799 mg, 1.05 equiv.) in THF (40 mL) was added EEDQ (*N*‐ethoxycarbonyl‐2‐ethoxy‐1,2‐dihydroquinoline; 1.60 g, 1.05 equiv.) and the resulting mixture was stirred for 23 h at room temperature. The solvent was removed, and the residue was dissolved in AcOEt (200 mL). The organic layer was washed with 1m NaHCO_3_, 10 % aqueous citric acid, and brine (100 mL each), dried over Na_2_SO_4_, filtered, and evaporated. The resulting solid was again dissolved in AcOEt (200 mL) and treated as described above to yield an off‐white solid (3.26 g, 91 %). ^1^H‐NMR (MeOD): 1.34–1.43 (*m*, 12 H); 1.53–1.59 (*m*, 2 H); 1.72–1.81 (*m*, 1 H); 1.86–1.93 (*m*, 1 H); 2.88 (*dd*, *J*=14, 9, 1 H); 3.12–3.17 (*m*, 3 H); 4.36 (*dd*, *J*=9, 5, 1 H); 4.47 (*dd*, *J*=9, 5, 1 H); 4.52 (*d*, *J*=5, 2 H); 4.58 (*d*, *J*=7, 2 H); 5.18 (*dd*, *J*=11, 1, 2 H); 5.29 (*dd*, *J*=17, 2, 1 H); 5.89–5.96 (*m*, 1 H); 7.23–7.31 (*m*, 5 H); 7.34 (*d*, *J*=9, 2 H); 7.57 (*d*, *J*=9, 2 H).

**H‐Phe‐Lys(alloc)‐PABOH⋅HCl** (**4**). A stirred solution of **3** (3.26 g, 5.59 mmol) in EtOH (30 mL) was treated with HCl conc. (2.34 mL, 5 equiv.). After 20 h the solvent was evaporated, the residue was dissolved in a minimum amount of EtOH and precipitated by dropwise addition to AcOEt (120 mL). The resulting white solid was filtered off, washed with AcOEt and Et_2_O, and dried *in vacuo* (1.84 g, 63 %). ^1^H‐NMR (MeOD): 1.42–1.53 (*m*, 2 H); 1.55–1.61 (*m*, 2 H); 1.76–1.84 (*m*, 1 H); 1.88–1.94 (*m*, 1 H); 3.07 (*dd*, *J*=14, 8, 1 H); 3.14 (*t*, *J*=7, 2 H); 3.30–3.34 (*m*, 1 H); 4.20 (*dd*, *J*=8, 6, 1 H); 4.51–4.54 (*m*, 3 H); 4.60 (*s*, 2 H); 5.18 (*dd*, *J*=11, 1, 2 H); 5.30 (*dd*, *J*=17, 2, 1 H); 5.89–5.97 (*m*, 1 H); 7.25–7.31 (*m*, 5 H); 7.36 (*d*, *J*=9, 2 H); 7.58 (*d*, *J*=9, 2 H). ^13^C‐NMR (MeOD): 24.0; 30.6; 33.0; 38.6; 41.4; 55.4; 55.5; 64.8; 66.3; 117.4; 121.2 (2 C); 128.6 (2 C); 128.8; 130.1 (2 C); 130.5 (2 C); 134.5; 135.4; 138.6; 138.9; 158.9; 169.5; 171.7. ESI‐MS: 483.26 ([*M*+H]^+^).

**Morph‐Phe‐Lys(alloc)‐PABOH** (**5**). To a solution of **4** (500 mg, 0.963 mmol) and Et_3_N (134 μL, 1 equiv.) in EtOH (5 mL), morpholin‐4‐yl‐acetic acid (140 mg, 1 equiv.) and EEDQ (250 mg, 1.05 equiv.) were added. After stirring for 24 h at room temperature, the solvent was evaporated, the residue was treated with Et_2_O (5 mL), sonicated, and then left to stand for 2 h at room temperature. The solid was collected by filtration, washed with Et_2_O, and dried *in vacuo*. The crude product was purified by flash chromatography over silica gel, eluting with AcOEt/MeOH 10 : 1, to give the product as a white powder (283 mg, 48 %). ^1^H‐NMR (MeOD): 1.40–1.52 (*m*, 2 H); 1.53–1.60 (*m*, 2 H); 1.76–1.83 (*m*, 1 H); 1.89–1.96 (*m*, 1 H); 2.26–2.30 (*m*, 2 H); 2.37–2.41 (*m*, 2 H); 2.88 (*d*, *J*=16, 1 H); 3.02 (*dd*, *J*=14, 9, 1 H); 3.06 (*d*, *J*=16, 1 H); 3.14 (*t*, *J*=7, 2 H); 3.26 (*dd*, *J*=14, 6, 1 H); 3.60 (*t*, *J*=5, 4 H); 4.49 (*dd*, *J*=9, 6, 1 H); 4.52 (*d*, *J*=5, 2 H); 4.60 (*s*, 2 H); 4.77 (*dd*, *J*=9, 5, 1 H); 5.18 (*dd*, *J*=11, 1, 1 H); 5.30 (*dd*, *J*=17, 2, 1 H); 5.89 – 5.97 (*m*, 1 H); 7.18–7.19 (*m*, 1 H); 7.23–7.28 (*m*, 4 H); 7.35 (*d*, *J*=9, 2 H); 7.59 (*d*, *J*=9, 2 H). ^13^C‐NMR (MeOD): 24.1; 30.6; 32.9; 38.8; 41.5; 54.6 (2 C); 55.1; 55.4; 62.5; 64.9; 66.3; 67.9 (2 C); 117.4; 121.3 (2 C); 128.0; 128.6 (2 C); 129.6 (2 C); 130.4 (2 C); 134.6; 138.0; 138.6; 138.9; 158.9; 172.3; 172.6; 173.5. ESI‐MS: 610.26 ([*M*+H]^+^).

**Morph‐Phe‐Lys(alloc)‐PABC‐PNP** (**6**). A solution of **5** (283 mg, 0.464 mmol) and DIPEA (237 μL, 3 equiv.) in dry DMF (9.5 mL) was treated with bis(4‐nitrophenyl)carbonate (212 mg, 1.5 equiv.), and the mixture was stirred for 25 h at room temperature. Then, AcOEt (100 mL) was added and the organic layer was washed with 0.01 m KOH (10×30 mL) and brine (1×10 mL), dried over MgSO_4_, filtered, and concentrated *in vacuo*. The crude product was purified by flash chromatography over silica gel, eluting with AcOEt/MeOH 20 : 1, to give the product as a white foam (256 mg, 71 %). ^1^H‐NMR (MeOD): 1.42–1.52 (*m*, 2 H); 1.54–1.61 (*m*, 2 H); 1.77–1.84 (*m*, 1 H); 1.90–1.97 (*m*, 1 H); 2.26–2.30 (*m*, 2 H); 2.36–2.41 (*m*, 2 H); 2.88 (*d*, *J*=16, 1 H); 3.02 (*dd*, *J*=14, 9, 1 H); 3.06 (*d*, *J*=16, 1 H); 3.14 (*t*, *J*=7, 2 H); 3.27 (*dd*, *J*=14, 5, 1 H); 3.60 (*t*, *J*=5, 4 H); 4.49–4.52 (*m*, 3 H); 4.77 (*dd*, *J*=9, 5, 1 H); 5.17 (*dd*, *J*=11, 1, 2 H); 5.29 (*dd*, *J*=17, 2, 1 H); 5.30 (*s*, 2 H); 5.89–5.96 (*m*, 1 H); 7.18–7.21 (*m*, 1 H); 7.25–7.28 (*m*, 4 H); 7.47 (*d*, *J*=9, 2 H); 7.48 (*d*, *J*=9, 2 H); 7.68 (*d*, *J*=9, 2 H); 8.33 (*d*, *J*=9, 2 H). ESI‐MS: 775.47 ([*M*+H]^+^), 797.47 ([*M*+Na]^+^).

**Morph‐Phe‐Lys(alloc)‐PABC‐Sun** (**7**). To a stirred solution of **6** (330 mg, 0.426 mmol) in dry DMF (4.5 mL) were added 4‐DMAP (57 mg, 1.1 equiv.) and sunitinib (187 mg, 1.1 equiv.), and the mixture was stirred at room temperature in the dark. After 69 h AcOEt (100 mL) was added and the resulting mixture was stirred for another 5 min. The orange solid was filtered off, washed with small amounts of AcOEt, and dried *in vacuo* (295 mg, 67 %). ^1^H‐NMR (MeOD/CDCl_3_): 1.08 (*t*, *J*=7, 6 H); 1.36–1.44 (*m*, 2 H); 1.51–1.56 (*m*, 2 H); 1.70–1.78 (*m*, 1 H); 1.86–1.92 (*m*, 1 H); 2.24–2.28 (*m*, 2 H); 2.37–2.42 (*m*, 2 H); 2.49 (*s*, 3 H); 2.55 (*s*, 3 H); 2.62 (*q*, *J*=7, 4 H); 2.69 (*t*, *J*=6, 2 H); 2.86 (*d*, *J*=16, 1 H); 2.96–3.05 (*m*, 2 H); 3.12 (*t*, *J*=7, 2 H); 3.22 (*dd*, *J*=14, 5, 1 H); 3.48 (*t*, *J*=7, 2 H); 3.55–3.60 (*m*, 4 H); 4.46 (*dd*, *J*=8, 6, 1 H); 4.50 (*d*, *J*=5, 2 H); 4.69 (*dd*, *J*=8, 5, 1 H); 5.16 (*d*, *J*=10, 1 H); 5.26 (*d*, *J*=17, 1 H); 5.47 (*s*, 2 H); 5.84–5.92 (*m*, 1 H); 6.89 (*ddd*, *J*=11, 9, 2, 1 H); 7.16–7.23 (*m*, 5 H); 7.29 (*dd*, *J*=8, 2, 1 H); 7.47 (*s*, 1 H); 7.51 (*d*, *J*=9, 2 H); 7.64 (*d*, *J*=9, 2 H); 7.75 (*dd*, *J*=9, 4, 1 H). ^13^C‐NMR (MeOD/CDCl_3_): 11.1; 11.5 (2 C); 13.9; 23.3; 29.8; 32.2; 37.5; 38.4; 40.9; 47.4 (2 C); 52.3; 54.1 (2 C); 54.3; 54.6; 62.0; 65.9; 67.5 (2 C); 69.1; 104.9 (^*2*^ *J*_CF_=25); 112.7 (^*4*^ *J*_CF_=2); 113.6 (^*2*^ *J*_CF_=24); 116.9 (^*3*^ *J*_CF_=9); 117.5; 120.9 (2 C); 121.2; 125.7; 126.9; 127.6; 126.7; 128.0 (^*3*^ *J*_CF_=10); 129.2 (2 C); 129.7 (2 C); 130.2 (2 C); 131.5; 132.2; 133.3; 133.6; 136.9 ; 139.2; 139.6; 151.5; 160.9 (^*1*^ *J*_CF_=241); 167.1; 168.2; 171.4; 171.7; 172.5. ESI‐MS: 517.76 ([*M*+2 H]^2+^).

**Morph‐Phe‐Lys‐PABC‐Sun⋅3HCOOH** (**8**). A suspension of **7** (125 mg, 0.121 mmol) and 1,3‐dimethylbarbituric acid (21 mg, 1.1 equiv.) in dry DMF (4 mL) was treated with Pd(PPh_3_)_4_ (7 mg, 0.05 equiv.) and stirred for 30 min. Then, the solvent was evaporated. The residue was purified through RP‐HPLC [H_2_O/MeCN (0.1 % HCOOH); 20–61 % MeCN] to give the product as an orange solid (43 mg, 33 %). ^1^H‐NMR (MeOD): 1.19 (*t*, *J*=7, 6 H); 1.46–1.59 (*m*, 2 H); 1.67–1.75 (*m*, 2 H); 1.78–1.86 (*m*, 1 H); 1.92–1.99 (*m*, 1 H); 2.26–2.30 (*m*, 2 H); 2.35–2.40 (*m*, 2 H); 2.48 (*s*, 3 H); 2.51 (*s*, 3 H); 2.83 (*q*, *J*=7, 4 H); 2.87–2.95 (*m*, 5 H); 2.99–3.05 (*m*, 2 H); 3.23 (*dd*, *J*=14, 5, 1 H); 3.55–3.59 (*m*, 6 H); 4.54 (*dd*, *J*=9, 5, 1 H); 4.68 (*dd*, *J*=9, 5, 1 H); 5.46 (*s*, 2 H); 6.92 (*ddd*, *J*=9, 9, 3, 1 H); 7.16–7.20 (*m*, 1 H); 7.22–7.27 (*m*, 4 H); 7.50 (*dd*, *J*=9, 3, 1 H); 7.57 (*d*, *J*=9, 2 H); 7.61 (*s*, 1 H); 7.68 (*d*, *J*=9, 2 H); 7.78 (*dd*, *J*=9, 5, 1 H). ^13^C‐NMR (MeOD): 9.5; 9.7 (2 C); 12.1; 22.4; 26.9; 31.2; 36.2; 37.2; 39.2; 47.0 (2 C); 51.5; 53.2 (2 C); 53.6; 54.0; 61.0; 66.5 (2 C); 68.0; 104.3 (^*2*^ *J*_CF_=26); 112.0 (^*4*^ *J*_CF_=3); 112.5 (^*2*^ *J*_CF_=25); 115.8 (^*3*^ *J*_CF_=9); 119.9 (2 C); 120.3; 125.0; 126.2; 126.7; 127.4 (^*3*^ *J*_CF_=9); 128.3 (2 C); 129.0 (2 C); 129.2 (2 C); 131.3; 131.7; 132.7; 136.5; 138.5 (2 C); 150.8; 160.3 (^*1*^ *J*_CF_=241); 166.9; 167.3; 170.7; 171.3; 172.3. ESI‐MS: 475.75 ([*M*+2 H]^2+^), 950.49 ([*M*+H]^+^). Anal. calc. for C_51_H_64_FN_9_O_8_ ⋅ 3 HCOOH (*M*_r_=1088.18 g/mol): C 59.60, H 6.48 N 11.58; found: C 59.73, H 6.40, N 11.82. (See *Figure S1* and *S2*).

**Boc‐Gly‐Gly‐PABOH** (**9**). To a mixture of Boc‐Gly‐Gly‐OH (200 mg, 0.861 mmol) and PABOH (111 mg, 1.05 equiv.) in THF (3 mL) was added EEDQ (224 mg, 1.05 equiv.), and the resulting mixture was stirred for 24 h at room temperature. The solvent was removed, and the residue was dissolved in AcOEt (60 mL). The organic layer was washed with 1 m NaHCO_3_, 10 % aqueous citric acid, and brine (30 mL each), dried over Na_2_SO_4_, filtered, and evaporated to yield a slightly orange solid (187 mg, 64 %). ^1^H‐NMR (MeOD): 1.45 (*s*, 9 H); 3.77 (*s*, 2 H); 4.02 (*s*, 2 H); 4.56 (*s*, 2 H); 7.30 (*d*, *J*=8, 2 H); 7.58 (*d*, *J*=8, 2 H).

**H‐Gly‐Gly‐PABOH** (**11**). A stirred solution of **9** (187 mg, 0.554 mmol) in EtOH (50 mL) was treated with HCl conc. (231 μL, 5 equiv.). After 18 h the solvent was evaporated yielding H‐Gly‐Gly‐PABOH⋅HCl (**10**) as a slightly orange powder which was used for the next step without further purification (151 mg, 100 %). ^1^H‐NMR (MeOD): 3.81 (*s*, 2 H); 4.12 (*s*, 2 H); 4.59 (*s*, 2 H); 7.34 (*d*, *J*=9, 2 H); 7.56 (*d*, *J*=9, 2 H). A solution of **10** (70 mg, 0.26 mmol) in H_2_O (1 mL) was basified to pH 8 with sat. NaHCO_3_. After removal of the solvent, the crude product was purified by flash chromatography over silica gel, eluting with CH_2_Cl_2_/MeOH 6 : 1 (1 % Et_3_N), to give the product as an off‐white solid (26 mg, 43 %). ^1^H‐NMR (MeOD): 3.41 (*s*, 2 H); 4.08 (*s*, 2 H); 4.58 (*s*, 2 H); 7.33 (*d*, *J*=9, 2 H); 7.56 (*d*, *J*=9, 2 H).

**Morph‐Gly‐Gly‐PABOH** (**12**). To a solution of **11** (440 mg, 1.85 mmol) and DIPEA (947 μL, 3 equiv.) in dry DMF (30 mL), morpholin‐4‐yl‐acetic acid (296 mg, 1.1 equiv.) and TBTU (715 mg, 1.2 equiv.) were added. After stirring for 24 h at room temperature, the solvent was evaporated, and the crude product was purified by flash chromatography over silica gel, eluting with CH_2_Cl_2_/MeOH 16 : 1 (1 % Et_3_N). Further purification on aluminum oxide, eluting with CH_2_Cl_2_/MeOH 1 : 1, gave the product as a white powder (504 mg, 75 %). ^1^H‐NMR ((D_6_)DMSO): 2.47 (*t*, *J*=5, 4 H); 2.98 (*s*, 2 H); 3.62 (*t*, *J*=5, 4 H); 3.81 (*d*, *J*=6, 2 H); 3.90 (*d*, *J*=6, 2 H); 4.44 (*d*, *J*=6, 2 H); 5.11 (*t*, *J*=6, 1 H); 7.25 (*d*, *J*=9, 2 H); 7.56 (*d*, *J*=9, 2 H); 8.06 (*t*, *J*=6, 1 H); 8.27 (*t*, *J*=6, 1 H); 9.80 (*s*, 1 H).

**Morph‐Gly‐Gly‐PABC‐PNP** (**13**). A solution of **12** (100 mg, 0.274 mmol) and DIPEA (152 μL, 3 equiv.) in dry DMF (3 mL) was treated with bis(4‐nitrophenyl)carbonate (125 mg, 1.5 equiv.), and the mixture was stirred for 24 h at room temperature. Then, AcOEt (100 mL) was added and the organic layer was washed with 0.01 m KOH (10×30 mL), dried over MgSO_4_, filtered, and concentrated *in vacuo*. The crude product was purified by flash chromatography over silica gel, eluting with CH_2_Cl_2_/MeOH 15 : 1, to give the product as a white foam (112 mg, 77 %). ^1^H‐NMR ((D_6_)DMSO): 2.47 (*t*, *J*=5, 4 H); 2.99 (*s*, 2 H); 3.62 (*t*, *J*=5, 4 H); 3.82 (*d*, *J*=6, 2 H); 3.92 (*d*, *J*=6, 2 H); 5.26 (*s*, 2 H); 7.44 (*d*, *J*=9, 2 H); 7.58 (*d*, *J*=9, 2 H); 7.67 (*d*, *J*=9, 2 H); 8.07 (*t*, *J*=6, 1 H); 8.30 (*t*, *J*=6, 1 H); 8.33 (*d*, *J*=9, 2 H); 9.96 (*s*, 1H).

**Morph‐Gly‐Gly‐PABC‐Sun** (**14**). To a stirred solution of **13** (62 mg, 0.12 mmol) in dry DMF (2.5 mL) were added 4‐DMAP (16 mg, 1.1 equiv.) and sunitinib (50 mg, 1.1 equiv.), and the mixture was stirred at room temperature in the dark. After 72 h, AcOEt (40 mL) was added and the resulting mixture was stirred for another 10 min. The orange solid was filtered off, washed with small amounts of AcOEt, and dried *in vacuo* (38 mg, 41 %). ^1^H‐NMR ((D_6_)DMSO): 0.98 (*t*, *J*=7, 6 H); 2.45–2.55 (*m*, 16 H); 2.97 (*s*, 2 H); 3.29 (*dt*, *J*=7, *J*=6, 2 H); 3.60 (*t*, *J*=5, 4 H); 3.80 (*d*, *J*=6, 2 H); 3.91 (*d*, *J*=6, 2 H); 5.41 (*s*, 2 H); 7.05 (*ddd*, *J*=9, 9, 3, 1 H); 7.50 (*d*, *J*=9, 2 H); 7.60 (*t*, *J*=6, 1 H); 7.66 (*d*, *J*=9, 2 H); 7.75 (*dd*, *J*=9, 5, 1 H); 7.78 (*s*, 1 H); 7.93 (*dd*, *J*=9, 3, 1 H); 8.08 (*t*, *J*=6, 1 H); 8.32 (*t*, *J*=6, 1 H); 9.98 (*s*, 1 H); 12.75 (*s*, 1 H). ^13^C‐NMR ((D_6_)DMSO): 11.1; 12.3 (2 C); 13.8; 37.5; 42.3; 43.1; 47.0 (2 C); 52.1; 53.8 (2 C); 61.9; 66.6 (2 C); 68.4; 105.9 ( ^*2*^ *J*_CF_=26.3); 111.3 (^*4*^ *J*_CF_=2.7); 113.0 (^*2*^ *J*_CF_=24.0); 116.1 (^*3*^ *J*_CF_=8.9); 119.6 (2 C); 122.4; 126.3; 126.6; 128.0 (^*3*^ *J*_CF_=10.0); 129.6 (2 C); 130.6; 131.8; 133.6; 139.3; 139.4; 150.8; 160.0 (^*1*^ *J*_CF_=238.9); 164.6; 166.8; 168.3; 169.8; 170.3. ESI‐MS: 789.54 ([*M*+H]^+^). Anal. calc. for C_40_H_49_FN_8_O_8_ ⋅ H_2_O (*M*_r_=806.88 g/mol): C 59.54, H 6.37, N 13.89; found: C 59.55, H 6.33, N 13.76. (See *Figure S3* and *S4*).

### *Stability Measurements in Aqueous Buffer*

Compounds **8**, **14**, and sunitinib were dissolved in 10 mm sodium phosphate buffer (pH 7.4, 37 °C) or *Mc Coy*’s medium (+10 % fetal calf serum) containing 1 % DMSO at a concentration of 10 μm and incubated at 37 °C. Aliquots were withdrawn at different time points (0–24 h, every 44 min) and subjected to RP‐HPLC analysis. The HPLC chromatogram peak areas at 420 nm were used to calculate the concentration of the remaining prodrug. The sunitinib chromatograms were used as reference for the quantification of formed free drug. Unknown peaks were identified using mass spectrometry. (See *Figures S5–S10*).

### *Biology*

*Cathepsin B Cleavage Assay*. Cathepsin B (human liver, 0.47 mg/mL, 324 U/mg) was obtained from *Merck Millipore*, *Merck KGaA*. The following protocol was applied to the test compounds: 5 μL of cathepsin B stock were added to 10 μL of activation buffer (30 mm DTT/15 mm EDTA‐Na_2_ in H_2_O) and incubated for 15 min at room temperature. The solution was diluted with 1.5 mL of 25 mm sodium acetate buffer (1 mm EDTA, pH 5, preheated to 37 °C), and the reaction was initiated by the addition of 7 μL of compound stock solution (10 mm in MeOH; final concentration: *ca*. 46 μm). Aliquots were withdrawn and the reactions were followed by RP‐HPLC as a function of time. The HPLC chromatogram peak areas at 420 nm were used to determine both the disappearance of the starting material as well as the appearance of sunitinib. Between the time points, all solutions were incubated at 37 °C. Reference solutions containing 7 μL of compound stock solution, 1.5 mL of buffer solution, and 15 μL of activation buffer were prepared and analyzed under the same conditions and at the same time points in order to distinguish between cathepsin B‐based activation and enzyme‐free hydrolysis. A solution of sunitinib (prepared as described for the reference measurements) served as standards for the quantification of the reaction product.

*Chemicals for Cell Culture Tests*. Sunitinib malate (from *LC Laboratories*®) and all other investigated compounds were dissolved in DMSO. These stock solutions were further diluted into culture media at the indicated concentrations. The final DMSO concentrations were always less than 1 %.

*Cell Culture*. The cell models used in this study are summarized in *Table S1* together with their culture media as well as the respective sources. All cells were cultivated in humidified incubators (37 °C, 21 % O_2_, 5 % CO_2_) in the indicated culture medium containing 10 % fetal calf serum (*PAA*, Linz Austria). Cell cultures were periodically checked for *Mycoplasma* contamination.

*Cytotoxicity Assay*. Cells were plated (2×10^3^ cells/well) in 96‐well plates and allowed to recover for 24 h. Subsequently, the dissolved drugs were added. After 72 h of drug exposure, the proportion of viable cells was determined by MTT assay following the manufacturer's recommendations (*EZ4U*, *Biomedica*, Vienna, Austria). Cytotoxicity was expressed as *IC*_50_ values calculated from full dose‐response curves using Graph Pad Prism software.

*Western Blot Analysis*. To assess the impact of the tested compounds on receptor tyrosine kinase signaling, cells were seeded in 6‐well plates and (without further manipulation) treated with the compounds as indicated. For protein isolation, cells were harvested and lysed, and the protein extracts resolved by SDS/PAGE and transferred onto a polyvinylidene difluoride membrane for Western blotting as previously described.^33^ The details on the antibodies used are given in *Table S2*. Additionally, horseradish peroxidase‐labeled secondary antibodies from *Santa Cruz Biotechnology* were used at working dilutions of 1 : 10000.

*Gelatin Zymography*. Cathepsin B activity in total cell extracts was determined by gelatin zymography. To this end, 20 mg of the cell lysates were resolved by SDS/Page using 10 % SDS‐polyacrylamide gels containing 10 % gelatin (*Sigma−Aldrich*) under ice‐cooled conditions. The gels were washed four times in washing buffer [containing 2.5 % *Triton X‐100*, 50 mm sodium acetate, 100 mm sodium chloride (pH 5.5) and 10 mm l‐cystine] to remove the SDS. The gels were then incubated overnight in an incubation buffer containing 50 mm sodium acetate, 100 mm sodium chloride (pH 5.5), 10 mm l‐cystine and 5 mm EDTA. Next, the gels were stained with *Coomassie Blue R250* for 2 to 3 h. Gelatinolytic activities were identified as clear zones of lysis against a dark background. (See *Figures S11–S13*).

## Author Contribution Statement

*C. K*. and *M. M*. performed the synthetic experiments and analyzed the data. *B. K*. and *K. H*. performed the biological experiments and analyzed the data. *C. R. K*. and *P. H*. designed the experiments. *C. K*., *C. R. K*., *P. H*., *W. B*., and *B. K. K*. wrote/corrected the article.

## Supporting information

As a service to our authors and readers, this journal provides supporting information supplied by the authors. Such materials are peer reviewed and may be re‐organized for online delivery, but are not copy‐edited or typeset. Technical support issues arising from supporting information (other than missing files) should be addressed to the authors.

Supplementary
