# Supplementary material for: Synthesis, Characterization and in vitro Studies of a Cathepsin B‐Cleavable Prodrug of the VEGFR Inhibitor Sunitinib
Source: Chem Biodivers. 2018 Dec 19;16(1):e1800520. doi: 10.1002/cbdv.201800520 (PMC6391952; doi:10.1002/cbdv.201800520)
Supplement: Supplementary file 1 — Supplementary [file CBDV-16-na-s001.pdf]

CHEMISTRY & BIODIVERSITY

Supporting Information

© Copyright Wiley-VCH Verlag GmbH & Co. KGaA, 69451 Weinheim, 2019

Synthesis, Characterization and *in vitro* Studies of a Cathepsin B-Cleavable Prodrug of the VEGFR Inhibitor Sunitinib

Claudia Karnthaler-Benbakka, Bettina Koblmüller, Marlene Mathuber, Katharina Holste, Walter Berger, Petra Heffeter, Christian R. Kowol,* and Bernhard K. Keppler© 2019 The Authors. Published by Wiley-VCH AG. This is an open access article under the terms of the Creative Commons Attribution License, which permits use, distribution and reproduction in any medium, provided the original work is properly cited.

Supplementary Material

Synthesis, Characterization and In Vitro Studies of a Cathepsin B-cleavable Prodrug of the VEGFR Inhibitor Sunitinib

Claudia Karnthaler-Benbakka [a], Bettina Koblmüller [b], Marlene Mathuber [a], Katharina Holste [b], Walter Berger [b,c], Petra Heffeter [b,c], Christian R. Kowol [a,c,*], and Bernhard K. Keppler [a,c]

[a] *Institute of Inorganic Chemistry, University of Vienna, Waehringer Straße 42, 1090 Wien, Austria*

[b] *Institute of Cancer Research and Comprehensive Cancer Center, Medical University of Vienna, Borschkegasse 8A, 1090 Wien, Austria*

[c] *Research Platform "Translational Cancer Therapy Research", University of Vienna and Medical University of Vienna, Austria*

* Corresponding author. E-mail address: christian.kowol@univie.ac.at

List of Supplementary Material:

Scheme S1. Synthetic route of reference compound 14
Figure S1. ^{1}H NMR spectrum of compound 8
Figure S2. ^{13}C NMR spectrum of compound 8
Figure S3. ^{1}H NMR spectrum of compound 14
Figure S4. ^{13}C NMR spectrum of compound 14
Figure S5. Time curve of compound 8 in 10 mM phosphate buffer, 1% DMSO, pH 7.4, at 37°.
Figure S1. Time curve of compound 14 in 10 mM phosphate buffer, 1% DMSO, pH 7.4, at 37°.

Figure S2. Chromatograms of a stability study of compound 8 in 10 mM phosphate buffer, 1% DMSO, pH 7.4, 37°C, (A) recorded at 250 nm, and (B) recorded at the optimized wavelength for sunitinib, 420 nm.

Figure S3. Chromatograms of a stability study of compound 14 in 10 mM phosphate buffer, 1% DMSO, pH 7.4, 37°C, (A) recorded at 250 nm, and (B) recorded at the optimized wavelength for sunitinib, 420 nm.

Figure S4. Chromatograms comparing the formation of rearrangement product in 10 mM phosphate buffer (PB), 1% DMSO, pH 7.4, and Mc Coy's medium (medium) + 10% fetal calf serum, both 37°C, after 24 h for compound **8** at 420 nm.

Figure S5. Chromatograms comparing the formation of rearrangement product in 10 mM phosphate buffer (PB), 1% DMSO, pH 7.4, and Mc Coy's medium (medium) + 10% fetal calf serum, both 37°C, after 24 h for compound **14** at 420 nm.

Figure S6. Expression of sunitinib target RTKs in a selection of cancer cell lines used in the study. PDGFR β and VEGFR-1 expression of the indicated cell lines were measured in membrane-enriched fractions by Western blot analysis. β-actin expression served as loading control.

Figure S7. Cathepsin B expression levels of the cancer cell lines used in the study. Pro-cathepsin B and mature cathepsin B expression of the indicated cell lines were measured in cell extracts by Western blot analysis. β-actin expression served as loading control.

Figure S8. Comparison of the gelanolytic activities of cathepsin B in different cell lines. Cathepsin B activity was measured in cell extracts derived from the indicated cell lines by gelatin zymography.

Figure S9. The indicated cell lines were treated with the indicated concentrations of the test compounds. After 72 h treatment, cytotoxicity was determined by MTT assay. The values given are means plus standard deviations (SD) of one representative experiment (out of three) performed in triplicates.

Table S1. Overview of the used cell lines.

Table S2. Used antibodies.

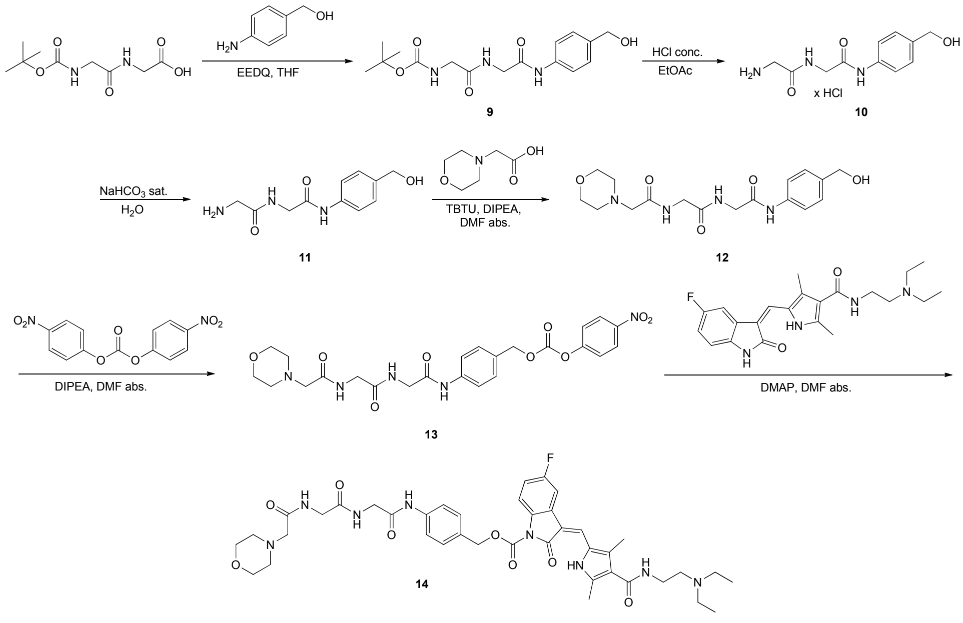

Scheme S1. Synthetic route of reference compound 14

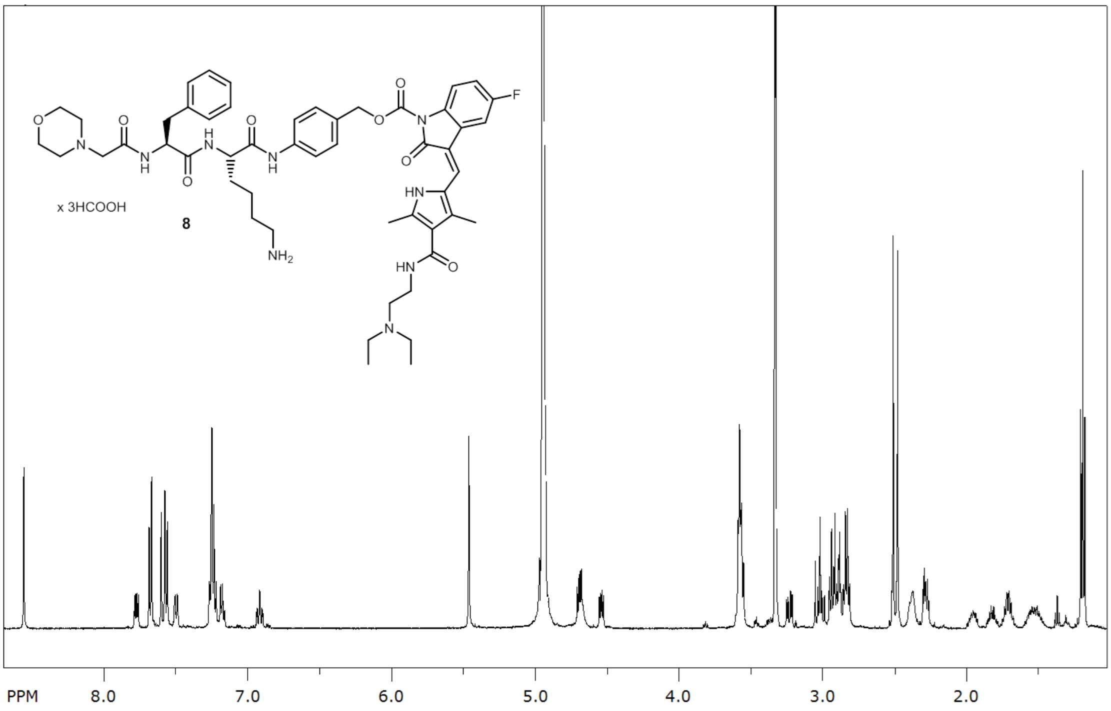

Figure S10. ^{1}H NMR spectrum of compound **8**.

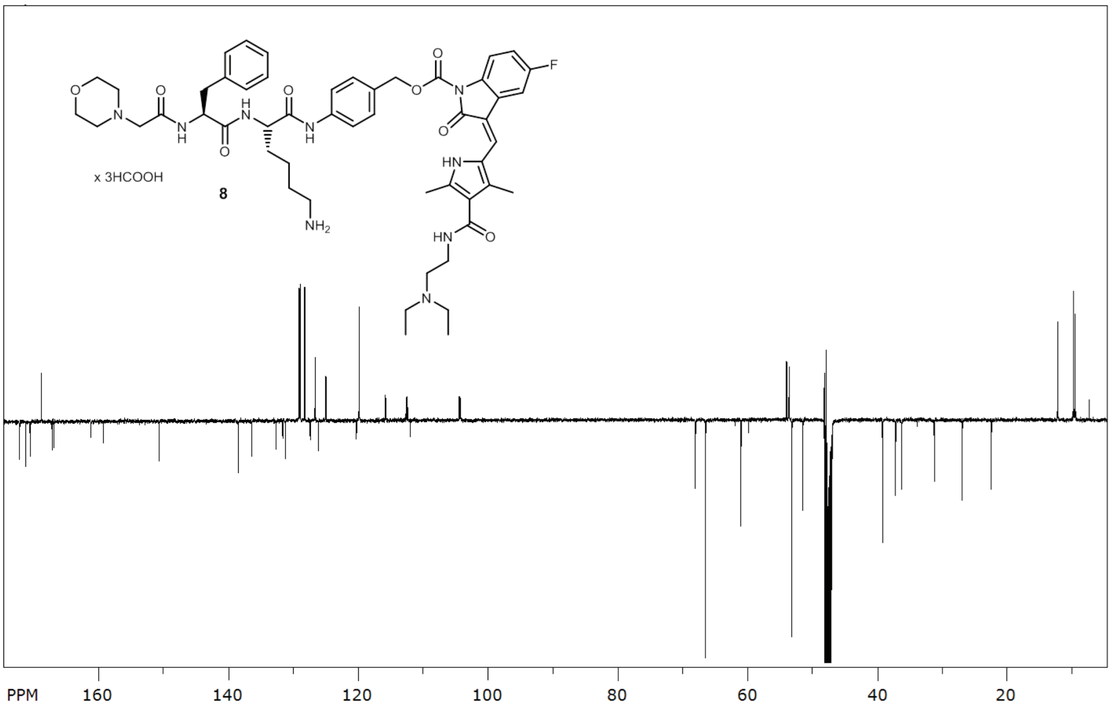

Figure S11. ^{13}C NMR spectrum of compound **8**.

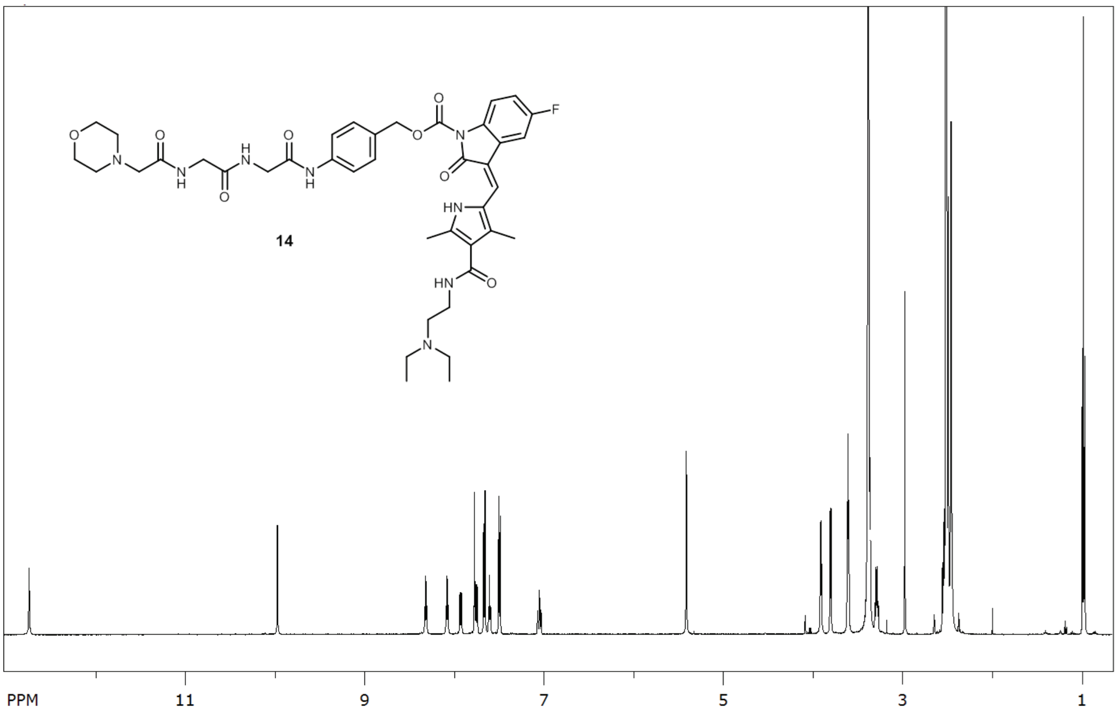

Figure S12. ^{1}H NMR spectrum of compound **14**.

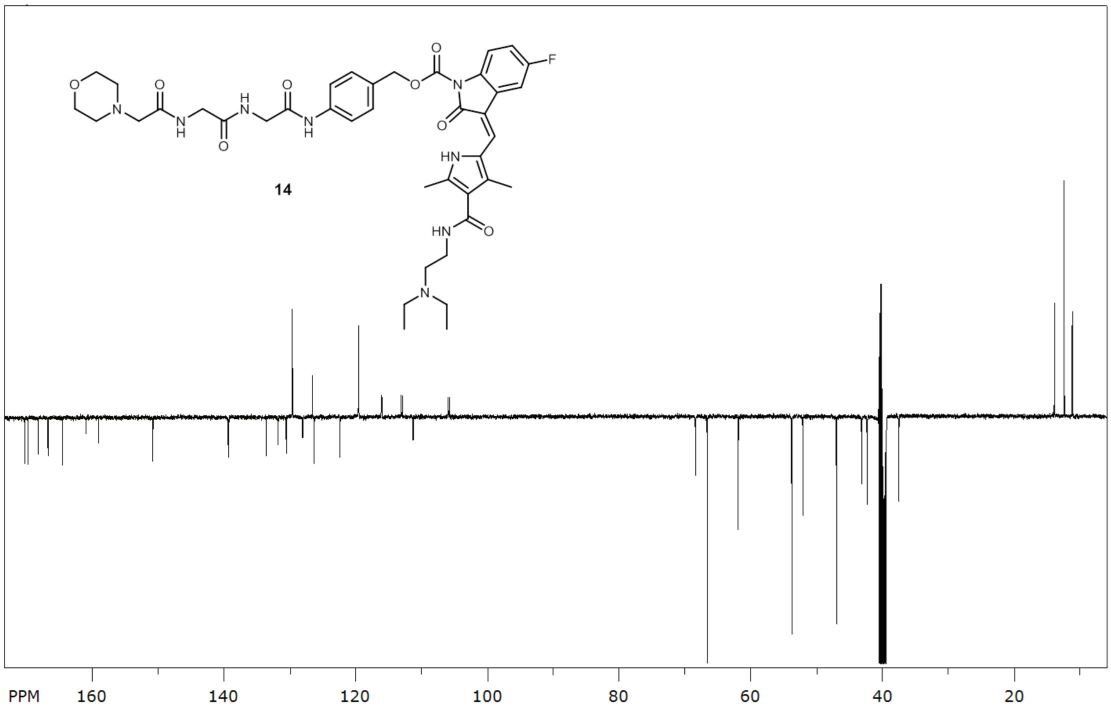

Figure S13. ^{13}C NMR spectrum of compound **14**.

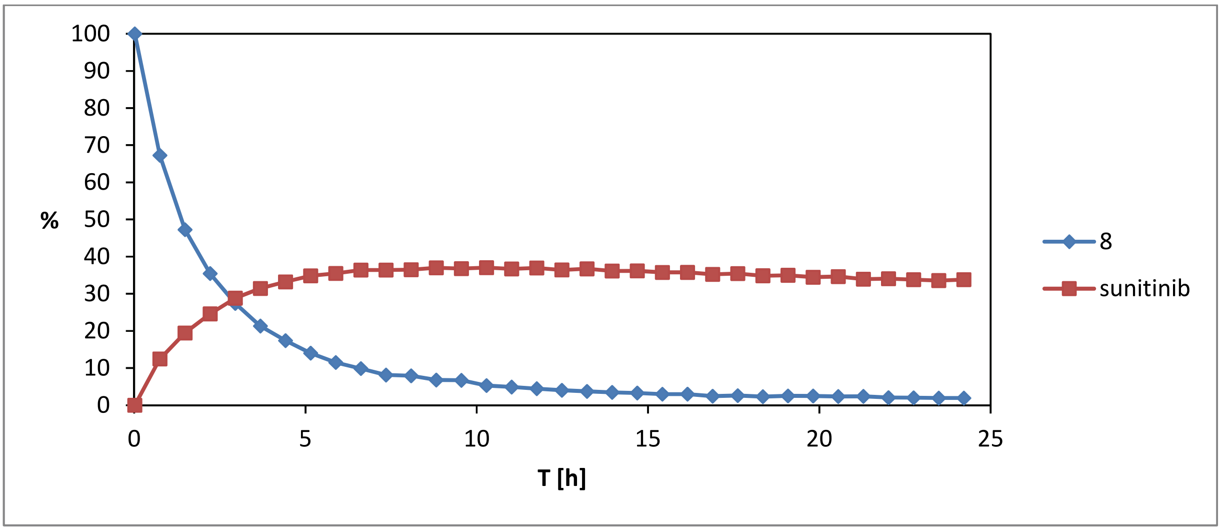

Figure S14. Time curve of compound **8** in 10 mM phosphate buffer, 1% DMSO, pH 7.4, at 37°.

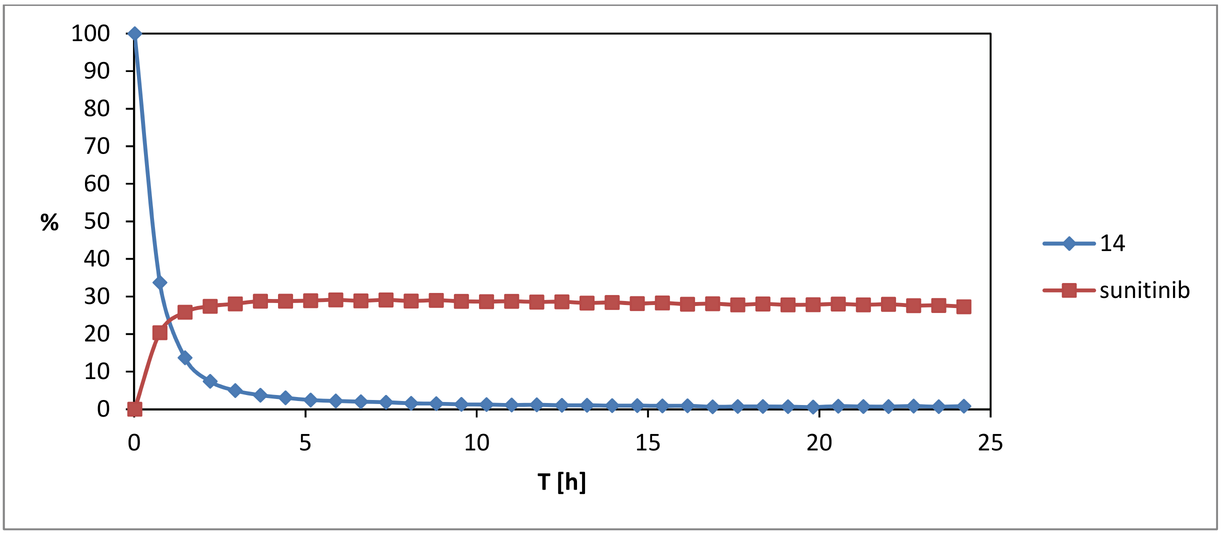

Figure S15. Time curve of compound **14** in 10 mM phosphate buffer, 1% DMSO, pH 7.4, at 37°.

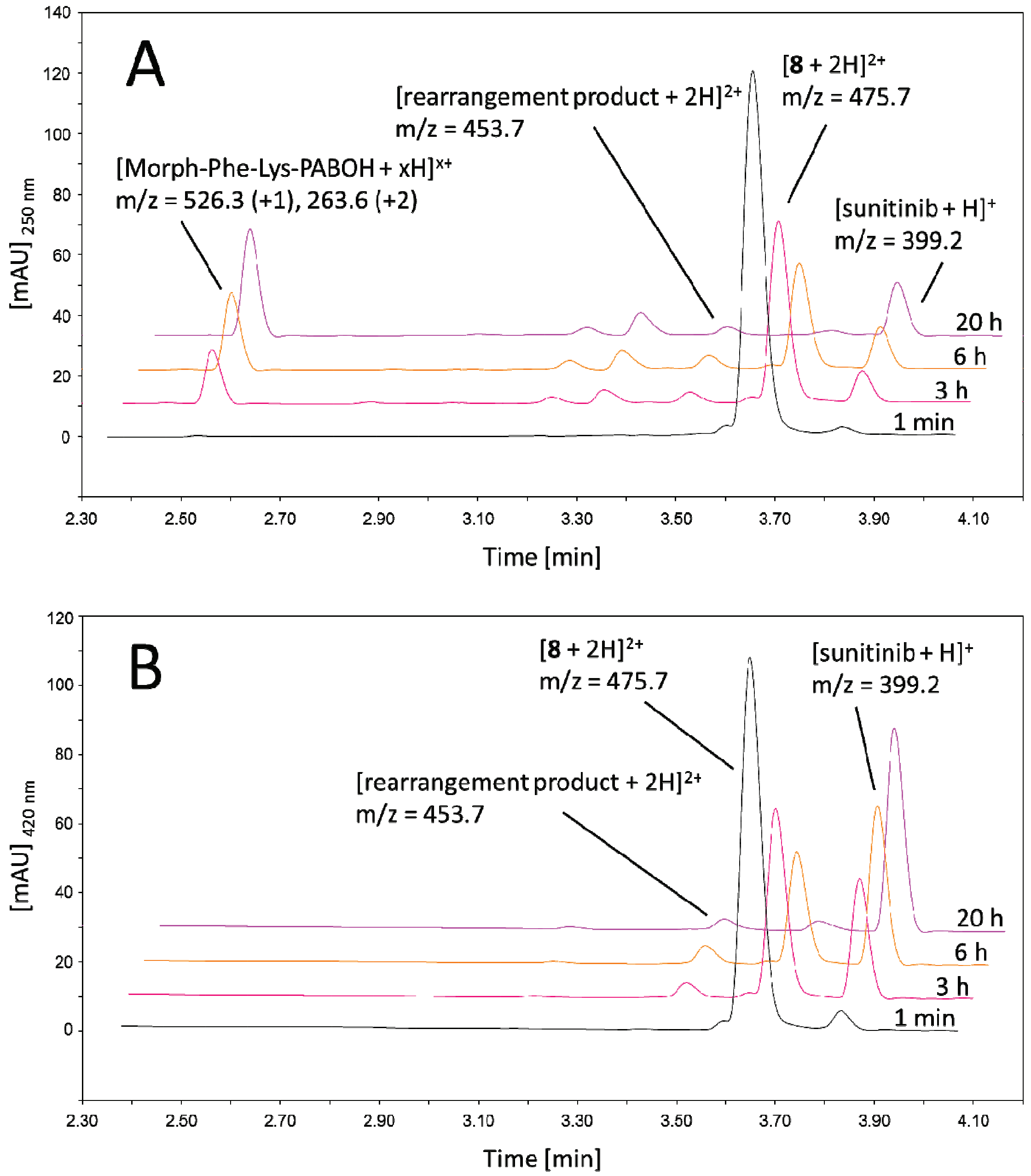

Figure S16. Chromatograms of a stability study of compound **8** in 10 mM phosphate buffer, 1% DMSO, pH 7.4, 37°C, **(A)** recorded at 250 nm, and **(B)** recorded at the optimized wavelength for sunitinib, 420 nm.

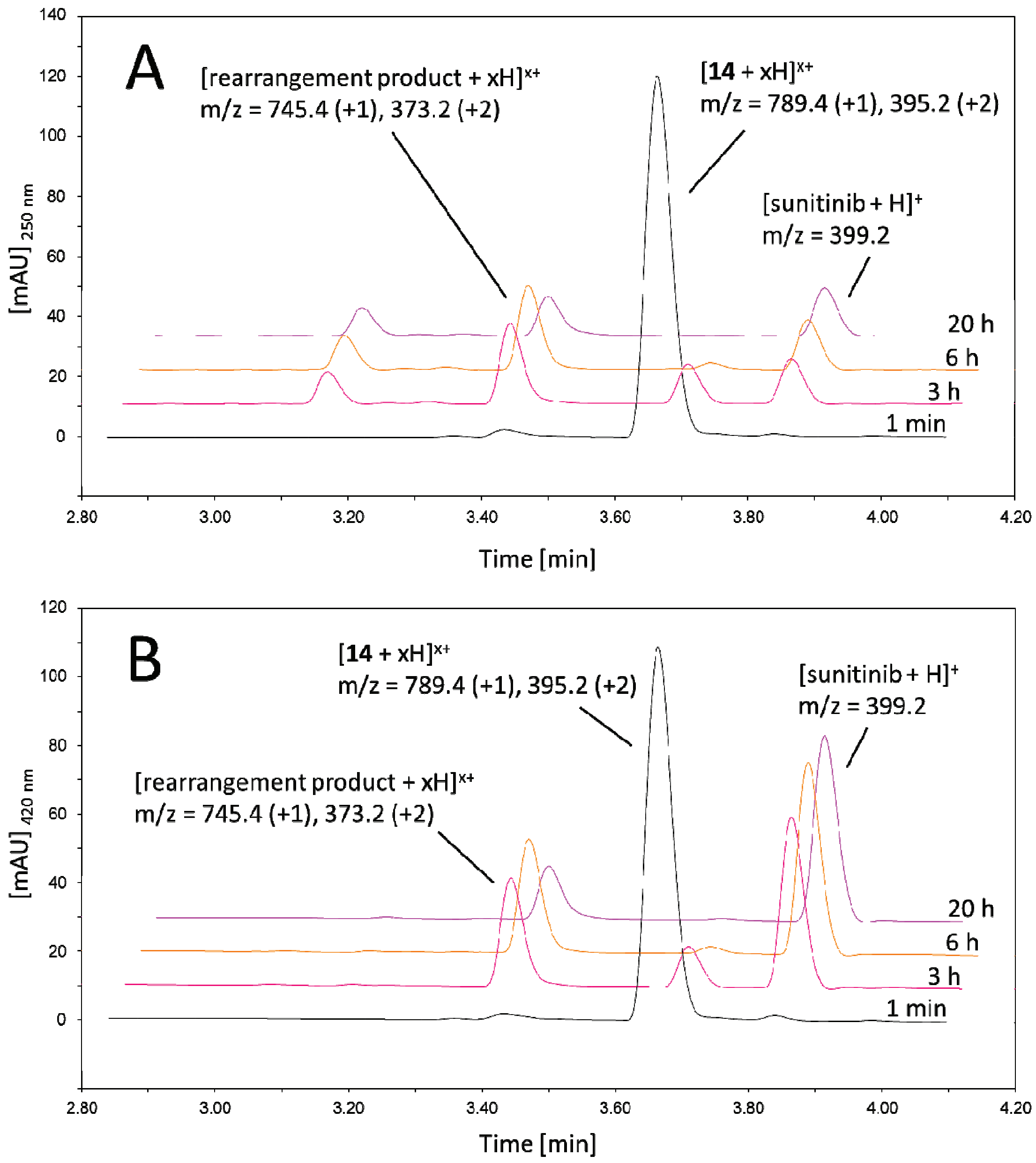

Figure S17. Chromatograms of a stability study of compound **14** in 10 mM phosphate buffer, 1% DMSO, pH 7.4, 37°C, **(A)** recorded at 250 nm, and **(B)** recorded at the optimized wavelength for sunitinib, 420 nm.

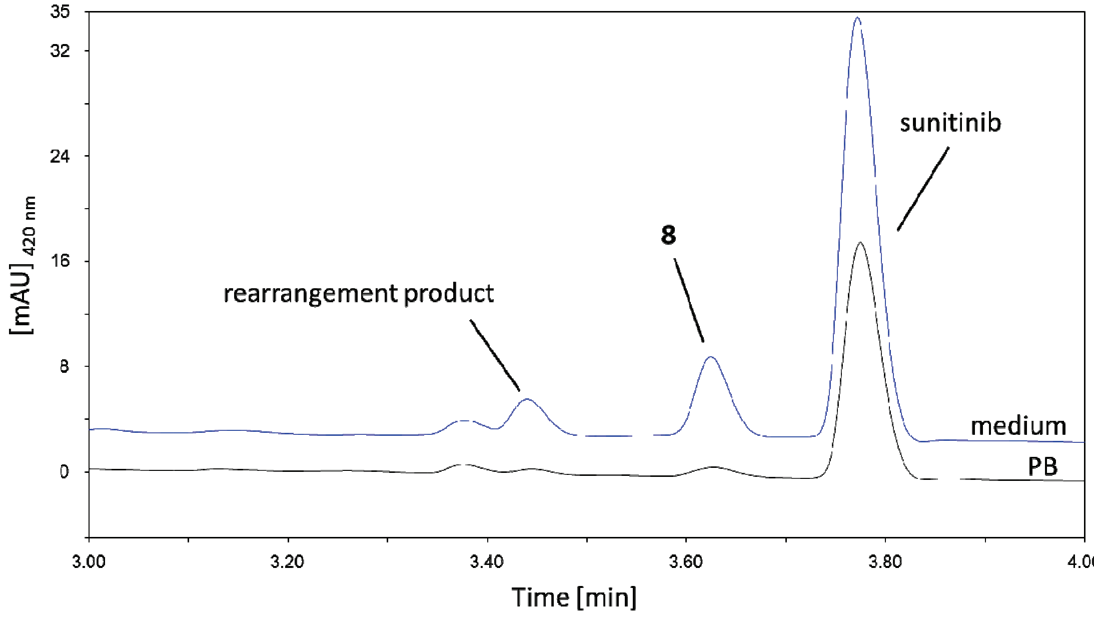

Figure S18. Chromatograms comparing the formation of rearrangement product in 10 mM phosphate buffer (PB), 1% DMSO, pH 7.4, and Mc Coy's medium (medium) + 10% fetal calf serum, both 37°C, after 24 h for compound **8** at 420 nm.

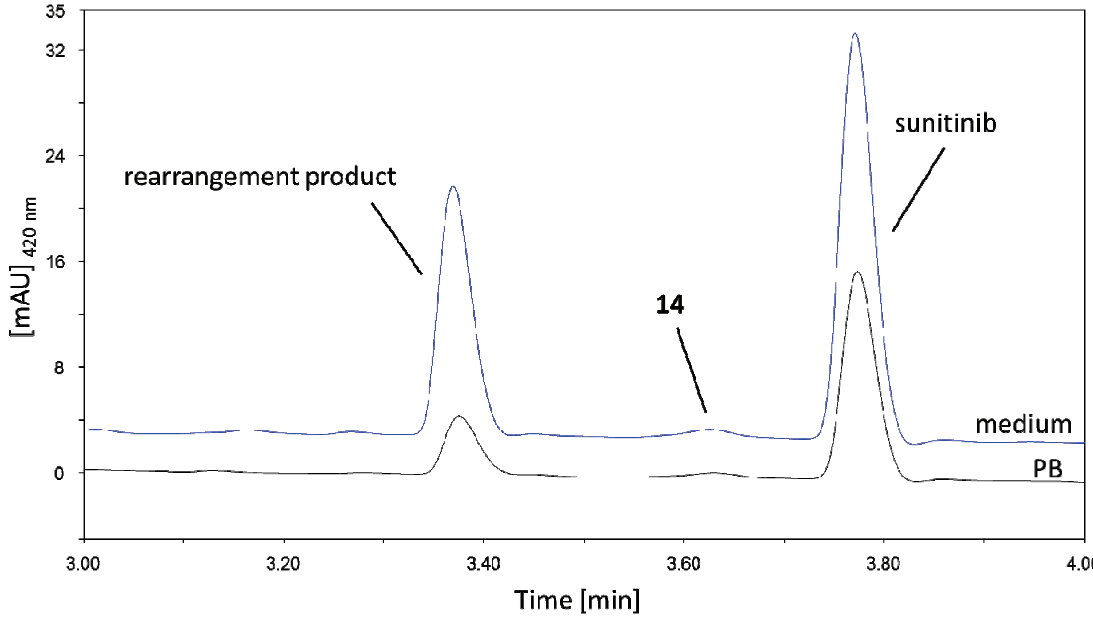

Figure S19. Chromatograms comparing the formation of rearrangement product in 10 mM phosphate buffer (PB), 1% DMSO, pH 7.4, and Mc Coy's medium (medium) + 10% fetal calf serum, both 37°C, after 24 h for compound **14** at 420 nm.

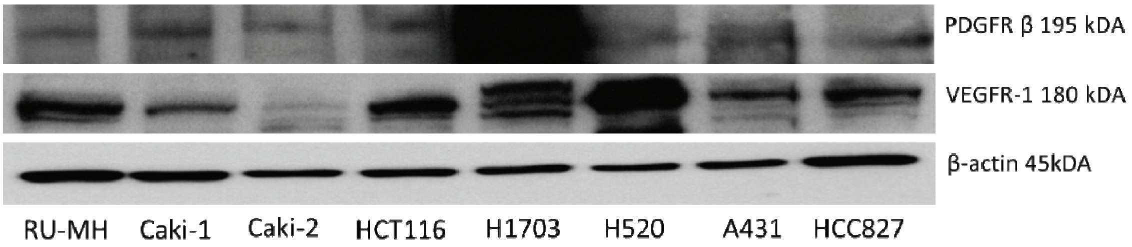

Figure S20. Expression of sunitinib target RTKs in a selection of cancer cell lines used in the study. PDGFR β and VEGFR-1 expression of the indicated cell lines were measured in membrane-enriched fractions by Western blot analysis. β-actin expression served as loading control.

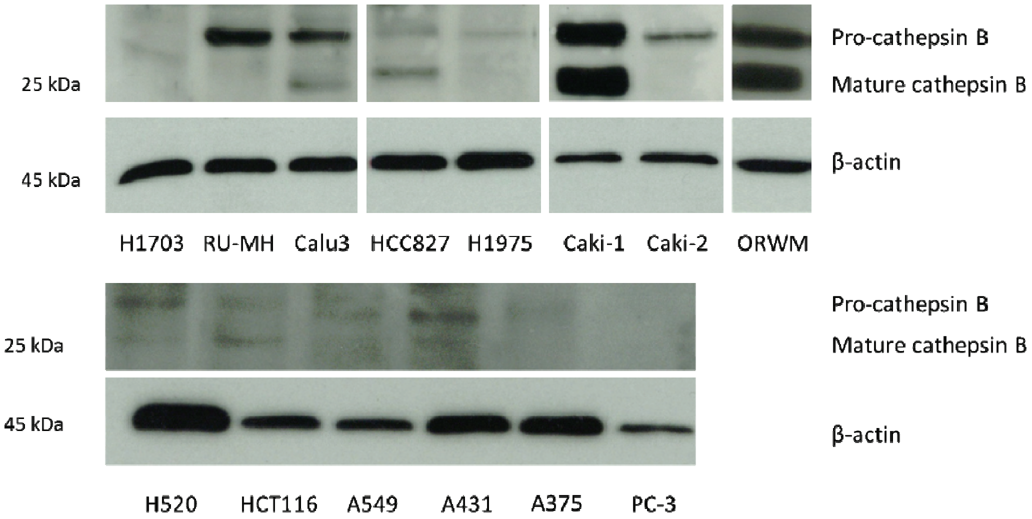

Figure S21. Cathepsin B expression levels of the cancer cell lines used in the study. Pro-cathepsin B and mature cathepsin B expression of the indicated cell lines were measured in cell extracts by Western blot analysis. β-actin expression served as loading control.

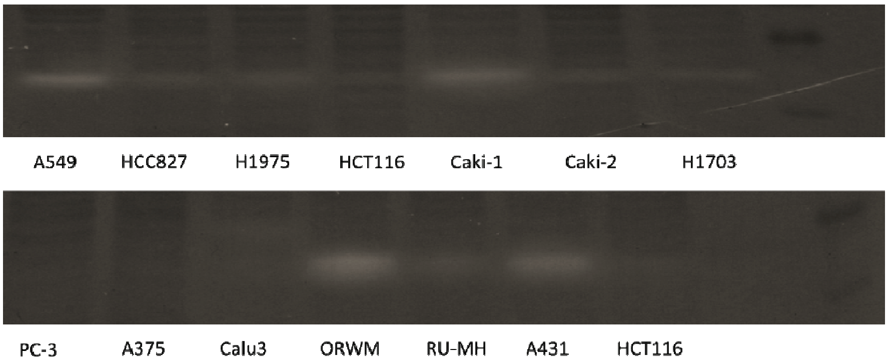

Figure S22. Comparison of the gelanolytic activities of cathepsin B in different cell lines. Cathepsin B activity was measured in cell extracts derived from the indicated cell lines by gelatin zymography.

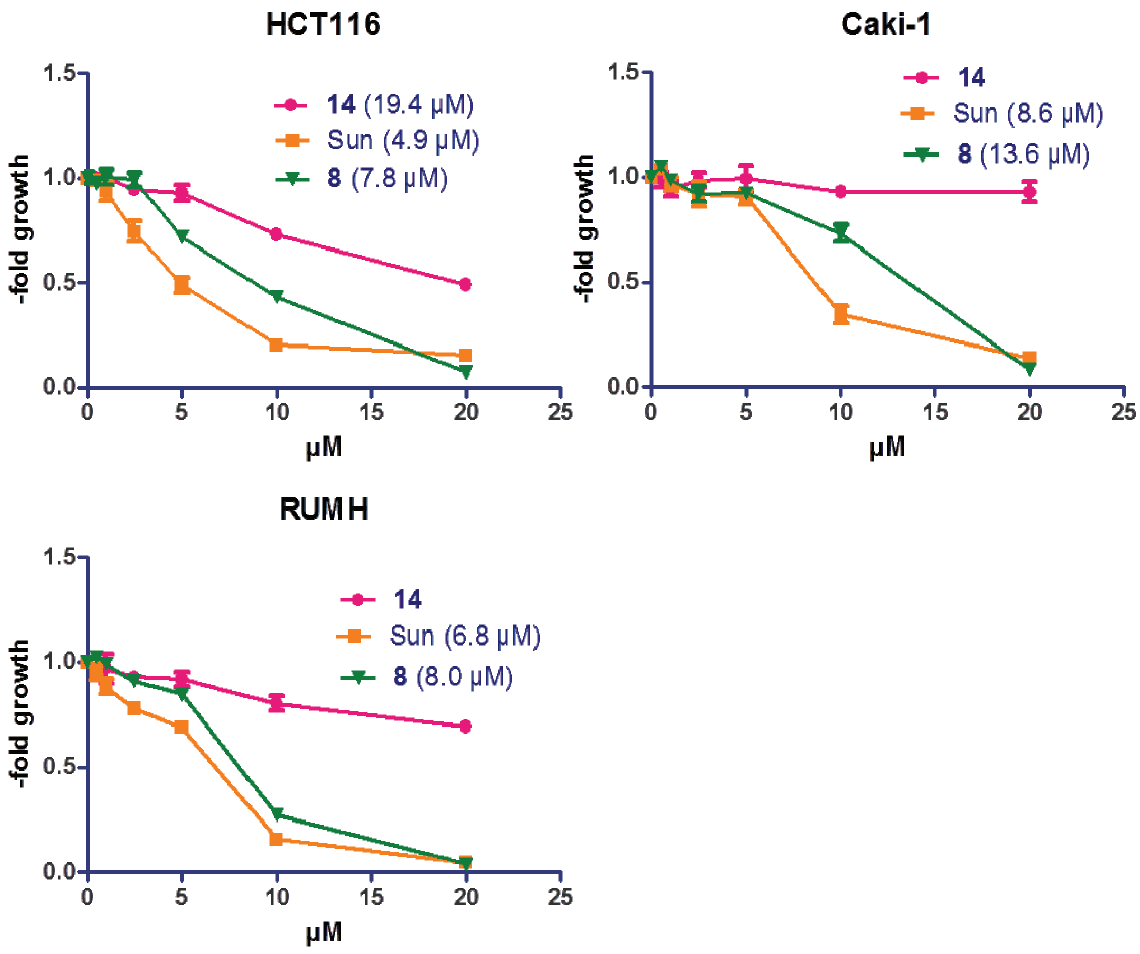

Figure S23. The indicated cell lines were treated with the indicated concentrations of the test compounds. After 72 h treatment, cytotoxicity was determined by MTT assay. The values given are means plus standard deviations (SD) of one representative experiment (out of three) performed in triplicates.

Table S3. Overview of the used cell lines.

Cell line	Histology	Growth medium	Source
Calu3	NSCLC, adenocarcinoma	MNP	ATCC
H1703	Squamous cell carcinoma of the lung	RPMI-1640	ATCC
H1975	NSCLC, adenocarcinoma	RPMI-1640	ATCC
HCC827	NSCLC, adenocarcinoma	RPMI-1640	ATCC
U87MG	Glioblastoma	DMEM	Prof. Filipic, Laibach
Caki-1	Renal cell carcinoma	McCoy's	ATCC
Caki-2	Renal cell carcinoma	McCoy's	ATCC
ORMW/8	Renal cell carcinoma	RPMI-1640	Established at ICR
RU-MH	Renal cell carcinoma	RPMI-1640	Established at ICR
A431	Epidermoid carcinoma	RPMI-1640	ATCC
A375	Melanoma	RPMI-1640	ATCC
PC-3	Prostate adenocarcinoma	RPMI-1640	ATCC
HCT116	Colorectal carcinoma	McCoy's	ATCC
A549	NSCLC, adenocarcinoma	RPMI-1640	ATCC
H520	Squamous cell carcinoma of the lung	RPMI-1640	ATCC

Abbreviations: ATCC, American Type Culture Collection Manassas VA; DMEM, Dulbecco's Modified Eagle's Medium; ICR, Institute for Cancer Research Vienna; MNP, Minimal Essential Medium with non-essential amino acids and pyruvate; NSCLC, Non-small cell lung cancer.

Table S4. Used antibodies.

Primary antibody	Specification	Dilution	Source
β-Actin	monoclonal mouse	1:5000	Sigma
Cathepsin B	Polyclonal goat	1:1000	Santa Cruz
VEGFR-1	Polyclonal rabbit	1:1000	Cell Signaling
PDGFR β	Monoclonar rabbit	1:1000	Cell Signaling
ERK 1/2 (p44/42 MAPK)	Polyclonal rabbit	1:1000	Cell Signaling
pERK (Thr202/Tyr204)	Polyclonal rabbit	1:1000	Cell Signaling
